# CDK4/6 Inhibitor PD0332991 in Glioblastoma Treatment: Does It Have a Future?

**DOI:** 10.3389/fonc.2015.00259

**Published:** 2015-11-30

**Authors:** Lisette B. W. Schröder, Kerrie L. McDonald

**Affiliations:** ^1^Erasmus Medical Center, Erasmus University Rotterdam, Rotterdam, Netherlands; ^2^Cure Brain Cancer Foundation Biomarkers and Translational Research Laboratory, Prince of Wales Clinical School, University of New South Wales, Kensington, NSW, Australia

**Keywords:** glioblastoma, PD0332991, palbociclib, Rb1, CDK4/6, blood–brain barrier

## Abstract

Glioblastoma is aggressive, highly infiltrating, and the most frequent malignant form of brain cancer. With a median survival time of only 14.6 months, when treated with the standard of care, it is essential to find new therapeutic options. A specific CDK4/6 inhibitor, PD0332991, obtained accelerated approval from the Food and Drug Administration for the treatment of patients with advanced estrogen receptor-positive and HER2-negative breast cancer. Common alterations in the cyclin D1-cyclin-dependent kinase 4/6-retinoblastoma 1 pathway in glioblastoma make PD0332991 also an interesting drug for the treatment of glioblastoma. Promising results in *in vitro* studies, where patient derived glioblastoma cell lines showed sensitivity to PD0332991, gave motive to start *in vivo* studies. Outcomes of these studies have been contrasting in terms of PD0332991 efficacy within the brain: more research is necessary to conclude whether CDK4/6 inhibitor can be beneficial in the treatment of glioblastoma.

## Introduction

Glioblastoma (*GBM, Astrocytoma grade IV*) is an aggressive, highly infiltrating form of brain cancer. Glioblastoma accounts for 15.6% of all primary brain tumors and, representing 45.2%, it is the most frequent malignant brain tumor (1, 2). However, glioblastoma is an uncommon disease. With an incidence rate of 3.19 per 100,000 in the US, it occurs over 21 times less often than breast cancer (2, 3).

Today, the standard of care for glioblastoma consists of surgical resection followed by radiotherapy in combination with temozolomide (4). Significant improvements to the standard treatment leading to overall survival extension have been lacking. This devastating disease has a median survival time of only 14.6 months (4). It is, therefore, essential to find new therapeutic options. This review focuses on the data on PD0332991 (*Palbociclib*) studies in glioblastoma and how this drug can play a role in glioblastoma treatment.

## The Cell Cycle

**Figure 1:**
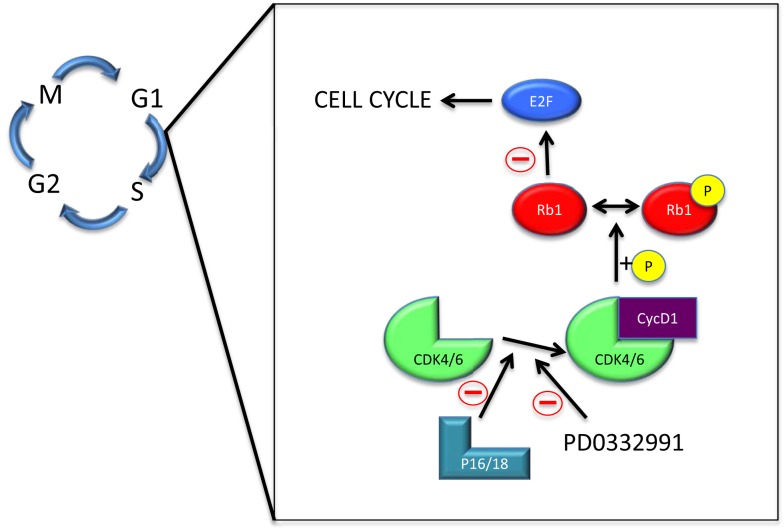
**The cyclin D1-cyclin-dependent kinase 4/6-retinoblastoma 1 pathway**. Abbreviations: M – mitosis; G1 – gap 1; S – synthesis; G2 – gap 2; Rb1 – retinoblastoma 1; CDK4/6 – cyclin-dependent kinase 4/6; cycD1 – cyclin D1; P – phosphate group.

The normal cell cycle consists out of four phases, known as G1, S, G2, and M. Checkpoints regulate progression to the next phase or initiate apoptosis (5). The cyclin D1-cyclin-dependent kinase 4/6-retinoblastoma 1 (*cycD1-CDK4/6-Rb1*) signaling pathway covers an important checkpoint in the cell cycle (6). It regulates G1 progression to S phase by a cascade of (in)activations (7) (Figure 1).

The main players in the cycD1-CDK4/6-Rb1 pathway are CDK4/6, p16, p18, cycD1, Rb1, and E2F. Free CDK4/6 can be inactivated by p16/18, or bind to cycD1 (8–11). The CDK4/6-cycD1 complex phosphorylates the negative growth regulator Rb1 to inhibit it from binding transcription factor E2F (12). Free E2F can then ultimately cause cell division (13).

The importance of CDK4/6 in cell cycle control makes it an interesting target for cancer treatment (14). Alterations in cell’s DNA can lead to cancer. These alterations cause cell division to be dysregulated, whereby the cell’s mechanisms are not able to control cell growth. Hyperactivity and amplification of CDK4/6, even as their genomic instability, are mechanisms in which the origin of cell cycle dysregulation can be found (15).

## PD0332991

PD0332991 is a highly specific CDK4/6 inhibitor (16) developed by Pfizer for cancer treatment (17). The drug inhibits CDK4/6, leading to inhibition of Rb1 phosphorylation and eventually to cell cycle blockage (18). PD0332991 has been a target of cancer research studies, especially within the field of breast cancer (19, 20). In 2004, Pfizer launched the first phase I clinical trial (ClinicalTrials.gov ID: NCT00141297). This trial was, in 2008, followed by a phase I/II clinical trial, named PALOMA-1, focusing on breast cancer treatment (21, 22). This clinical trial showed that PD0332991, as an addition to the breast cancer drug letrozole, significantly improved progression free survival for patients with advanced estrogen receptor-positive and HER2-negative breast cancer (22). Based on the results of this trial, PD0332991 obtained accelerated approval from the Food and Drug Administration (FDA) in the USA in 2015. The approval was specific to this subgroup of patients with breast cancer (23). Meanwhile, phase III trials for PD0332991 usage in breast cancer and phase I and II trials for PD0332991 treatment in other types of cancer have started (Table 1).

**Table 1:** **Clinical trials for PD0332991**.

Phase	Type of cancer	Name	ClinicalTrials.gov ID
Phase I	Colorectal	–	NCT01522989
	Central nervous system	–	NCT02255461
Phase I/II	Lung	–	NCT02022982
	Head and neck	–	NCT02101034
Phase II	Ovarian	–	NCT01536743
	Prostate	–	NCT02059213
	Glioblastoma	–	NCT01227434
Phase III	Breast	PALOMA-2	NCT01740427
		PALOMA-3	NCT01942135
		PALOMA-4	NCT02297438
		PENELOPE-B	NCT01864746
		PEARL	NCT02028507

## Rb1 Predicts Response to PD0332991

Preclinical studies on PD0332991 in breast cancer have shown that Rb1 functioning is the determining factor for the efficacy of treatment (24–27). Rb1 deficiency or loss of its function results in PD0332991 resistance (24–27). Dean and colleagues, in 2010, compared Rb1-deficient to Rb1-proficient cell lines for differences in PD0332991 treatment response, whereby, in most cell lines, knockdown of Rb1 resulted in failure to stop proliferation through CDK4/6 inhibition (24). Consistent with these results, Roberts and colleagues observed *in vivo* that PD0332991 did not reduce tumor growth in mice harboring Rb1-deficient tumors (26). Witkiewicz and colleagues reconfirmed these outcomes with the *in vitro* testing of PD0332991 on Rb1 knockdown and control cells (27). In line with these results, it has been shown that changes in the cycD1-CDK4/6-Rb1 pathway, leading to elevated expression of Rb1, are most sensitive to PD0332991 treatment (28). These changes include higher levels of cycD1 and Rb1 and lower levels of p16 (28). Presumably, p16 and Rb1 can be used as markers to predict response to PD0332991 (25).

The Cancer Genome Atlas Research Network (TCGA) revealed in 2008 that the cycD1-CDK4/6-Rb1 pathway is, with alterations in 78.9% of glioblastoma, among the top three most altered pathways (29, 30). The *p15/16* location, within this pathway, is the most common location for alteration (61%) (30). Other alterations found within the cycD1-CDK4/6-Rb1 pathway are *CDK4/6* (15.5%), *Rb1* (7.6%), *p18* (5.6%), and *cyclins* (2%) (30). Most of these alterations promote the pathway, which makes this a suitable aim for therapy.

In 2010, Michaud and colleagues published the first article on PD0332991 use in glioblastoma (31). Through *in vitro* experiments, they revealed the same strengths and weaknesses of this drug as was found in breast cancer: PD0332991 inhibited cell proliferation in Rb1 proficient glioblastoma cells and Rb1 deficiency caused resistance (31). In addition, codeletion of p16 and p18 was, by Wiedemeyer and colleagues, found to be another predictor of increased sensitivity (32). A remarkable outcome of this study was that high levels of CDK4 or CDK6 had no influence on the sensitivity to PD0332991 (32). This data was supported with a later study by Cen and colleagues who further suggested that *CDK4* amplification can be a sign of high resistance, while *CDK6* amplification can be a sign of high sensitivity (33). Based on the genetic profiles of patient material, Verhaak and colleagues were able to define a classification, creating subtypes for glioblastoma (34). Codeletion of p16/18 was described to be highly associated with one of the subtypes: classical glioblastoma (34). It will be interesting to see in future experiments whether the classical subtype of glioblastoma gain most benefit from treatment with PD0332991.

In order to be an effective drug for glioblastoma, PD0332991 needs to have the ability to reach the tumor and thereby the ability to cross the blood–brain barrier (*BBB*). Studies have shown that PD0332991 is capable of crossing the BBB, but there are conflicting ideas on the effectivity of PD0332991 within the brain (31, 33, 35–37).

In 2010, Michaud and colleagues demonstrated that at a dose of 150 mg/kg/day, PD0332991 acted as an effective antiproliferative drug intracranially (31). The survival analysis, where survival time of PD0332991 treated mice was significantly extended compared to vehicle control mice, confirmed these findings (31). A study by Cen and colleagues strongly supported these findings by reconfirming the antiproliferative effect of PD0332991 *in vivo* and the survival benefit of treatment (33).

Recently, various research groups have revisited PD0332991 and its effects within the brain. *In vitro* experiments showed that PD0332991 is a substrate of P-glycoprotein (P-gp) and breast cancer resistance protein (BCRP), both efflux transporters in the BBB (35–38). Through *in vivo* experiments, de Gooijer and colleagues as well as Parrish and colleagues showed that P-gp and BCRP cause limited PD0332991 delivery to the brain, resulting in low drug concentrations (35, 36). Treated with 150 mg/kg/day, the restricted PD0332991 brain penetration was shown to be inadequate to reach antiproliferative effects or survival benefits (36). It is not clear as to why the research groups have come to different conclusions in regard to the ability of PD0332991 to cross the blood–brain barrier. Patient-derived cell lines were intracranially injected into immunocompromised all of the studies and the same concentration of PD0332991 was used (150 mg/kg/day). Further studies are warranted.

To determine the efficacy of PD0332991, the University of California in San Francisco had started a phase II clinical trial in patients with Rb1 positive, recurrent glioblastoma (Table 1). In December 2013, after 23 patients had been enrolled in this trial, the investigators completed collecting their final data for the primary outcome measure. We are still eagerly waiting for the results of this clinical trial.

Overall, PD0332991 appears to be an interesting drug for glioblastoma treatment and could be a valuable addition to the standard of care. Raub and colleagues have tested PD0332991 in combination with temozolomide and unpublished data from McDonald’s lab has shown significant efficacy using this CDK 4/6 inhibitor in combination with radiotherapy. *In vivo* studies with this combination have started. Although there is some irregularity in the outcomes concerning PD0332991 crossing the BBB, *in vitro* results are promising. New *in vivo* experiments, advised in combination with P-gp and BCRP inhibitors, are recommended before more clinical trials on glioblastoma patients start. The potential of PD0332991 in the treatment of glioblastoma is encouraging to continue investigating PD0332991 in the hope of ultimately finding a cure for this devastating disease.

## Author Contributions

LS conducted the scientific review and wrote the manuscript. KM reviewed the manuscript.

## Conflict of Interest Statement

The authors declare that the research was conducted in the absence of any commercial or financial relationships that could be construed as a potential conflict of interest.
